# Enzymatic Deastringent Fruit Powder of Sea Buckthorn (*Hippophae rhamnoides* L.): Preparation, Antioxidant Activity Investigation and Metabolomics Analysis

**DOI:** 10.3390/foods15122240

**Published:** 2026-06-21

**Authors:** Jiaxuan Xie, Liting Lin, Haoran Yang, Daren Wu, Zhengxiao Zhang, Shan Lin, Feng Kang, Lingyu Zhang, Jian Li

**Affiliations:** 1 College of Ocean Food and Biological Engineering, Jimei University, Xiamen 361021, China; 202361000186@jmu.edu.cn (J.X.); xiaoyee1999@163.com (L.L.); 202321092093@jmu.edu.cn (H.Y.); darrenwu@jmu.edu.cn (D.W.); zxzhang@jmu.edu.cn (Z.Z.); 202161000080@jmu.edu.cn (S.L.); 2 Institute of Agro-Products Storage and Processing, Xinjiang Academy of Agricultural Sciences, Urumqi 830091, China; kf829167@126.com; 3 Fujian Provincial Engineering Technology Research Center of Marine Functional Food, Xiamen 361021, China

**Keywords:** sea buckthorn, enzymolysis, deastringency, antioxidant activity, metabolomics analysis

## Abstract

Sea buckthorn is a valuable medicinal and edible plant, but the sour and astringent taste of its fruit limits the development of the related processing industry. This study focused on establishing the optimal composite enzymatic hydrolysis strategy to reduce acerbity during processing of sea buckthorn berries, while preserving the antioxidant activity. The results indicated that the most effective conditions for deacidification and deastringency were achieved with a pectinase-to-tannase mass ratio of 1:1, an enzyme dosage of 0.20%, at a pH of 4.50, a temperature of 50 °C, and a duration of 4 h. Under this treatment, the sea buckthorn could retain its potent antioxidant activity. Furthermore, a significant alteration was observed in the levels of 36 metabolites, which were correlated with the sensory attributes of the sea buckthorn. The findings of this study provided a theoretical basis for the enhanced utilization of sea buckthorn in processing and for a deeper understanding of its bioactive properties.

## 1. Introduction

Sea buckthorn (SBT, *Hippophae rhamnoides* L.) is a deciduous shrub in the Elaeagnaceae family, primarily distributed along the countries of the Silk Road [1]. The sea buckthorn tree, known for its drought resistance and robust vitality, plays a crucial role in environmental conservation by preventing wind erosion and stabilizing sand dunes [2]. Moreover, it possesses significant edible and medicinal properties [3]. Sea buckthorn is packed with high levels of nutritional and bioactive compounds, like vitamins, polyphenols, sugars, and organic acids [4]. Sea buckthorn berry is widely recognized for the exceptionally high vitamin C content, which is several times higher than that of many vitamin C-rich fruits such as lemon and blackberries [5]. In addition to vitamin C, the fruit contains nine free amino acids essential to human nutrition [6], and high oil content, which makes it an excellent source of unsaturated fatty acids [7]. Moreover, sea buckthorn berry boasts an impressive array of flavonoids, such as isorhamnetin, quercetin, kaempferol, etc. [8]. This diverse nutritional profile endows it with a range of health benefits, including antioxidant activity, mitigation of cardiovascular diseases, tumor suppression, and allergy reduction [9,10,11,12,13,14].

Sea buckthorn is also a plant with significant promise in the economic sphere. Since the 1940s, Russian scientists have developed a series of sea buckthorn foods and radiation protection creams for Russian cosmonauts [15]. Nowadays, with the in-depth studies on sea buckthorn bioactive properties and processing technology, the potential of sea buckthorn in the human food industry has aroused the interest of researchers and producers worldwide [16]. However, the consumption of fresh sea buckthorn berries is somewhat restricted due to the sour and astringent taste, which also to some extent hinders the growth of the associated processing industry. The organic acids, flavonoids and sugars are the primary contributors to its taste, playing a significant role in the fruit’s sensory characteristics [17,18]. According to previous studies, the sourness in sea buckthorn berries mainly stems from the high levels of malic acid, and the astringency is primarily due to phenolic compounds, like tannins and gallic acids [9,19]. Upon ingestion of sea buckthorn products, phenolic hydroxyl groups interact with amine groups in oral salivary proteins and polysaccharides, leading to protein denaturation and precipitation [20]. This interaction diminishes saliva’s lubricating capacity for oral mucosa, causing it to wrinkle and produce an astringent sensation [21,22].

Currently, the primary methods for reducing the astringency of fruit juice include physical adsorption, chemical combination, and biological enzymatic hydrolysis. Among these, biological enzymatic hydrolysis stands out as an efficient, gentle and green method for astringency removal. This process utilizes specific enzymes to break down astringent compounds, effectively reducing the astringency. Generally, a well-designed composite enzyme system allows for a broader range of substrates to be targeted, increasing the overall efficiency of the process. For juice processing, pectinase has been widely used to decompose pectin substances, enhancing juice yield and clarification while causing phenolic compounds to precipitate due to the absence of pectin’s protective influence [23]. Previous studies have used pectinase to enzymatically hydrolyze sea buckthorn fruits in order to increase the juice yield while retaining the bioactive substances [24,25]. Tannase can hydrolyze the tannins (the primary contributors to astringency) and release low-molecular-weight polyphenolic compounds, which play an important role in antioxidant properties and sensory qualities stability [20,26]. The use of a pectinase and tannase composite enzyme system not only improves the taste profile of the juice, but also has minimal impact on its nutritional content, making it an ideal approach for astringency reduction in fruits. For example, in the deastringency process of cashew apple juice, a 1% addition level of pectinase achieved a juice clarity of 95%, and 0.5% tannase led to a 52.5% reduction in tannin concentration [27]. Persimmon pulp is susceptible to re-astringency during the drying process. To resolve the issue, tannase and pectinase were employed to alleviate the sour and astringent taste. The resulting enzyme-treated dried persimmons exhibited a lower degree of tannin polymerization and galloylation, successfully eliminating astringency while preserving the fruit’s original flavor [28]. In the processing of NFC blueberry juice, the addition of 0.747% tannase and 0.066% pectinase complex increased the anthocyanin content in the juice to (656 ± 0.8) mg/L. After enzymatic hydrolysis, the contents of major volatile flavor compounds such as esters, alkenes, and ketones in the blueberry juice significantly increased, enhancing the aroma and nutritional value of the juice [29].

This study was to develop an effective enzyme-based treatment to reduce astringency in sea buckthorn while preserving its antioxidant activity as much as possible, and to investigate the key metabolites affecting sensory properties by analyzing changes in metabolites during the enzymatic hydrolysis process. The optimum composite enzymatic deacidification and deastringency process of sea buckthorn was determined by single-factor experiments and response surface methodology. The antioxidant potency of sea buckthorn post-enzymatic hydrolysis was evaluated on its performance across indicators of antioxidant efficacy (DPPH, ABTS, hydroxyl radical scavenging ability, and ferric reducing capacity), as well as the protective effect on a cellular model of oxidative stress damage. Moreover, based on UHPLC-Q-TOF MS untargeted metabolomics, further exploration was conducted on the differential metabolites, their correlations, and the metabolic pathways affected by the enzymatic hydrolysis process. The findings of this study may provide a feasible strategy for the intensive processing and high-value utilization of sea buckthorn and offer mechanistic insights into enzymatic improvement in functional fruit products.

## 2. Materials and Methods

### 2.1. Materials

Sea buckthorn fruits were provided by Xinjiang Sikai Food R&D Center (Co., Ltd.) (Changji, China). Food-grade pectinase (10,000 U/g, derived from *Aspergillus niger*) and tannase (100 U/g, derived from *Aspergillus niger*) were both purchased from Dongheng Huadao Biotechnology Co., Ltd. (Nanning, China). Analytical-grade reagents for DPPH, ABTS, total reducing power, and reducing power of hydroxyl radical were obtained from Sinopharm Chemical Reagent Co., Ltd. (Shanghai, China). DMEM high-sucrose medium was purchased from Hyclone (Logan, UT, USA). 0.25% Trypsin-EDTA was purchased from Gibco (New York, NY, USA). FBS was purchased from PAN-Biotech GmbH (Aidenbach, Germany). Acetonitrile was purchased from Merck (Darmstadt, Germany), and ammonium acetate was purchased from Sigma (Missouri, MO, USA).

### 2.2. Preparation of Lyophilized Sea Buckthorn Fruit Powder Post-Enzymatic Hydrolysis

Selected undamaged sea buckthorn fruits were washed three times with distilled water to eliminate surface dust. Afterward, at low temperatures, the fruits were processed with ultra-pure water in a 1:2 fruit-to-water weight ratio to extract the juice. The mixture was preliminarily filtered, yielding a crude sea buckthorn filtrate. Following the enzymatic hydrolysis parameters, a complex enzyme mixture was introduced into the filtrate to catalyze the hydrolysis reaction. Thereafter, the enzymes were inactivated by heating at 90 °C in a water bath for 10 min to terminate the enzymatic activity. This filtrate was subjected to centrifugal filtration at 4 °C and 8000 rpm to remove the oil layer and precipitate the residue, from which the clarified sea buckthorn pulp was obtained. The pulp was pre-frozen at −80 °C for 48 h before undergoing vacuum lyophilization for approximately 30 h to yield the freeze-dried sea buckthorn fruit powder post-enzymatic hydrolysis.

### 2.3. Determination of Total Phenol Content

Total phenol content was measured referring to the methodology of Matić et al. [30] with slight adjustments. Briefly, the standard solution of gallic acid, 0.5 mol/L Na_2_CO_3_ and 50 µg/mL of sea buckthorn solution were prepared using ultrapure water as solvent. The test solutions were mixed with Folinol solution and Na_2_CO_3_ solution in a volume ratio of 10:1:2, and the absorbance at 765 nm was measured after 60 min. The total phenol content of the samples was calculated using a standard curve generated from a concentration gradient of gallic acid.

### 2.4. Single Factor Experiments and Response Surface Methodology

Five factors, including enzyme complex ratio (pectinase/tannase, *w*/*w*), enzyme dosage, enzymolysis time, temperature and pH, were chosen to investigate their influences on total phenol content. Each factor was varied individually while maintaining the others at fixed levels.

Based on the results of the single-factor experiments, the sea buckthorn enzymatic hydrolysis process was further optimized by the Box–Behnken Design method [31]. A three-factor, three-level response surface method was used to determine the optimal enzymatic process conditions with total phenol content, response value, enzymatic pH, temperature and time as factors.

### 2.5. Electronic Tongue Grading and Sensory Evaluation

Add 90 mL of sea buckthorn pulp or its enzymatic hydrolysate samples to a special cup for the electronic tongue. The positive and negative electrodes and sensors were activated and calibrated 24 h in advance to ensure the stability of the sensors. Each sample was measured 4 times at room temperature, and the last 3 times of stable data were selected for subsequent analysis.

The study’s sensory evaluation team comprised 10 health-conscious researchers aged 18 to 45 from the beverage R&D lab, all with a basic grasp of food products and sharp sensory perception, free from detrimental habits. Each panelist tasted the samples individually and rated them based on the sensory score standard outlined in Table S1.

### 2.6. Determination of In Vitro Antioxidant Activity

To assess the total reducing capacity, sample solutions of varying concentrations (1 mL) were combined with 1% potassium ferricyanide (1 mL) and PBS buffer (2.5 mL), then incubated at 50 °C for 20 min. The reaction was stopped by introducing 0.1% ferric chloride (1.2 mL) and 10% TCA (2 mL). The subsequent absorbance at 700 nm was measured after 30 min. Ascorbic acid was used as a positive control in all the above antioxidant assays.

The hydroxyl radical scavenging capacity was evaluated by creating a reaction mixture with equal parts of a 9 mM salicylic acid ethanol solution, a 9 mM aqueous ferrous sulfate solution, an 8.8 mM aqueous hydrogen peroxide solution, and the sample solution. After a 30 min reaction period, the absorbance of the mixture at 510 nm was measured.

The DPPH radical scavenging ability was calculated by mixing the sample with a 0.4 mM DPPH working solution in equal proportions for 30 min and measuring the absorbance at 517 nm.

To evaluate the ABTS radical scavenging ability, varying concentrations of the test sample solution (10 µL) were combined with 190 µL of the ABTS working solution and mixed thoroughly. After a 30 min reaction period, the absorbance of the mixture at 734 nm was measured.

### 2.7. Determination of Cellular Protection Effect Against Oxidative Stress

LO2 cells were cultured in DMEM high-glucose medium (containing 10% fetal bovine serum and 1% antibiotics) and grown at 37 °C and 5% CO_2_ in a humidified incubator. The experiment was set up with a blank control group, a control group (PBS buffered salt solution), an H_2_O_2_ injury model group (1.2 mM), and protection groups with different concentrations of samples (1%), with six replicate wells in each group. The cells were inoculated at a density of 1.5 × 10^4^ cells per well in 96-well plates and cultured overnight. They were then exposed to various concentrations of the samples for 24 h, followed by the addition of medium containing 1.2 mM H_2_O_2_ for a 4 h incubation period. Further, 100 µL DMEM medium containing 0.5 mg/mL MTT was added and cells were incubated in a CO_2_ incubator in the dark for 4 h. The medium was removed and Formazan crystals formed by the cells were dissolved using 150 µL of DMSO. The absorbance was read at 490 nm using 630 nm as the reference wavelength.

### 2.8. Non-Targeted Metabolomics

#### 2.8.1. Sample Preparation

The samples underwent extraction using a pre-cooled mixture of methanol, acetonitrile, and water (2:2:1, *v*/*v*) via low-temperature ultrasonication for 30 min. Afterward, it was centrifuged at 14,000× *g* for 20 min at 4 °C following a 10 min stand at −20 °C. The supernatant was filtered and subjected to vacuum drying. For LC-MS analysis, 100 μL of an aqueous acetonitrile solution (1:1, *v*/*v*) was added to redissolve the dried supernatant, which was then recentrifuged at 14,000× *g* for 15 min at 4 °C.

#### 2.8.2. Chromatographic Conditions

An Agilent 1290 Infinity LC (UHPLC) (Agilent, Santa Clara, CA, USA) equipped with a C–18 column was utilized for analysis. The mobile phases consisted of solvent A (water with 25 mM ammonium acetate and 0.5% formic acid) and solvent B (100% acetonitrile). The column was set at 40 °C, with a flow rate of 0.4 mL/min, and the injection volume was 2 μL, with samples randomized at 4 °C. The gradient elution program is as follows: 0~0.5 min, 5% B; 0.5~10.0 min, 5~100% B (linear variation); 10.0~12.0 min, 100% B; 12.0~12.1 min, 100~5% B (linear variation); 12.1~16 min, 5% B.

#### 2.8.3. Q-TOF Mass Spectrometry Conditions

High-resolution mass spectrometry data acquisition was performed in IDA mode using a high-resolution mass spectrometry AB Triple TOF 6600 mass spectrometer. Following chromatographic separation, the ESI source conditions were set as follows: Ion Source Gas 1 (Gas 1), 60; Ion Source Gas 2 (Gas 2), 60; Curtain Gas (CUR), 30; source temperature, 600 °C; Ion Spray Voltage Floating (ISVF), ±5500 V (applied in both positive and negative modes). TOF MS scan *m*/*z* range was 60–1000 Da, and the product ion scan *m*/*z* range was 25–1000 Da. The accumulation time was 0.20 s/spectrum for the TOF MS scan and 0.05 s/spectrum for the product ion scan. MS/MS spectra were acquired by information-dependent acquisition (IDA) operated in high-sensitivity mode, with a declustering potential (DP) of ±60 V (for both positive and negative modes) and a collision energy (CE) of 35 ± 15 eV. The IDA parameters were set to exclude isotopes within 4 Da and to monitor 10 candidate ions per cycle.

### 2.9. Statistical Analysis

Each experiment was repeated three times independently, and the results are presented as “mean ± SD”. The data were analyzed using IBM SPSS Statistics 27.0.1. Significance was determined using *t*-tests, one-way ANOVA, and post hoc comparisons were performed using Dunnett. The normal distribution of the samples was verified by the Shapiro–Wilk test. Differences with *p* < 0.05 were considered statistically significant.

## 3. Results and Discussion

### 3.1. Single Factor Experiments for Sea Buckthorn Post-Enzymatic Hydrolysis

#### 3.1.1. Effects of the Enzyme Complex Ratio (Pectinase/Tannase, *w*/*w*)

**Figure 1:**
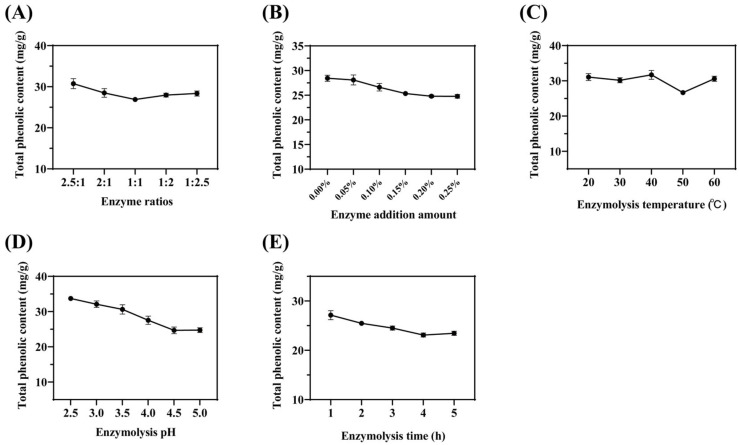
Effect of different enzymatic process parameters on the total phenolic content of sea buckthorn. (**A**) Composite enzyme with different mass ratios (pectinase: tannase). (**B**) Enzyme addition amount (*w*/*w*). (**C**) Enzyme digestion temperature. (**D**) Enzyme digestion pH. (**E**) Enzyme digestion time.

Pectinase breaks down the structural components of plant cell walls to facilitate tannin release, while tannase catalyzes the hydrolysis of ester linkages within tannins, yielding glucose and gallic acid [32]. An appropriate ratio helps to maximize the reduction in astringency in sea buckthorn. As shown in Figure 1A, the total phenol content initially decreased and then slightly increased with the increase in the tannase ratio, suggesting that a balanced decomposition between the two enzymes and their substrates was gradually achieved. The total phenol content was found to be at its lowest (26.89 ± 0.20 mg/g) when the pectinase-to-tannase ratio was 1:1. Consequently, this ratio has been selected as the optimal mass ratio for the composite enzyme.

#### 3.1.2. Effects of Enzyme Dosage

The effect of enzyme concentrations on the total phenol content of sea buckthorn was depicted in Figure 1B. Total phenol content exhibited a decreasing trend with increasing enzyme concentrations from 0.00% to 0.20%. At 0.25% enzyme concentration, the content plateaued, indicating complete enzymatic digestion of the substrate. Although the substrate could be fully digested at an enzyme concentration of 0.25%, the excessive enzyme dosage may cause ineffective consumption of enzyme preparations and fail to bring a better modification effect. In fact, excessive pectinase dosage could inhibit the enzymatic reaction rate by reducing effective water concentration and molecular diffusion, while also increasing juice viscosity due to sugar release [33]. Considering hydrolysis efficiency and production costs, 0.20% was identified as the optimal concentration for the composite deastringent enzyme, which is comparable to the used concentration in enzymatic modification of tea juice [34].

#### 3.1.3. Effects of Temperature on Enzymatic Hydrolysis

Figure 1C shows the impact of enzymatic hydrolysis temperature on the total phenol content of sea buckthorn. A negligible change was observed below 40 °C, followed by a significant decrease and slight rebound within the 40 to 60 °C range. This pattern indicated that the 40 to 60 °C range was optimal for the enzymatic reaction. Elevated temperature can enhance enzyme activity by increasing molecular collision rates within the optimal range. Concurrently, it improves substrate accessibility by increasing cell wall permeability and accelerating molecular diffusion. These combined effects promote the degradation of pectin substances and the release of phenolic compounds [35]. However, at higher temperatures, it was noted that the enzymes became inactivated. Integrating these observations, to balance the enzymatic activity and thermal stability, the optimal enzymatic hydrolysis temperature range for sea buckthorn was identified as 40 to 60 °C.

#### 3.1.4. Effects of pH on Enzymatic Hydrolysis

Pectinase and tannase used for juice improvement are both acidic proteins. Previous studies have shown that pectinase activity increases with pH in the range of 3–5, and a pH near 5 is optimal for tannase [36,37,38]. In this study, the influence of pH on the total phenol content in sea buckthorn was shown in Figure 1D. A decline in total phenol content was observed as the pH rises from 2.5 to 4.5, with a plateau beyond this threshold, indicating a relative stabilization of phenolic compounds. The native pH of sea buckthorn juice was around 3.20, and significant deviations from this value could perturb the fruit’s acidic milieu, predisposing it to vitamin C degradation and oxidative browning. To preserve the physicochemical properties of the juice, it is imperative to confine the pH manipulation within a narrow range. Thus, the optimal pH range for enzymatic digestion of sea buckthorn was ultimately selected to be 4.0~5.0.

#### 3.1.5. Effects of Enzymatic Hydrolysis Time

The enzymatic hydrolysis process requires sufficient time. Especially for pectinase, insufficient hydrolysis time leading to incomplete reaction prevents complete breakdown of the cell wall and allows its regeneration, whereas excessive hydrolysis time may result in deterioration of the taste of the mixed fruit pulp and loss of heat-sensitive nutrients [39,40]. The impact of enzyme digestion duration on the total phenol content in sea buckthorn was shown in Figure 1E. An initial decrease followed by a stabilization trend was observed in the total phenol content as the digestion time was extended. This behavior could be attributed to the reduction in substrate during the enzymatic process. With a constant enzyme loading, the reaction rate is initially high owing to abundant substrate. Over time, the rate progressively slows down as the substrate is consumed and products accumulate. Once the majority of the substrate is hydrolyzed, a further increase in reaction time no longer leads to a noticeable decrease in substrate quantity. Considering processing time and efficiency in practical production scenarios, the optimal duration for enzyme digestion was chosen to fall within the interval of 4 to 5 h.

### 3.2. Optimization of Enzymatic Hydrolysis Parameters by Response Surface Methodology

**Figure 2:**
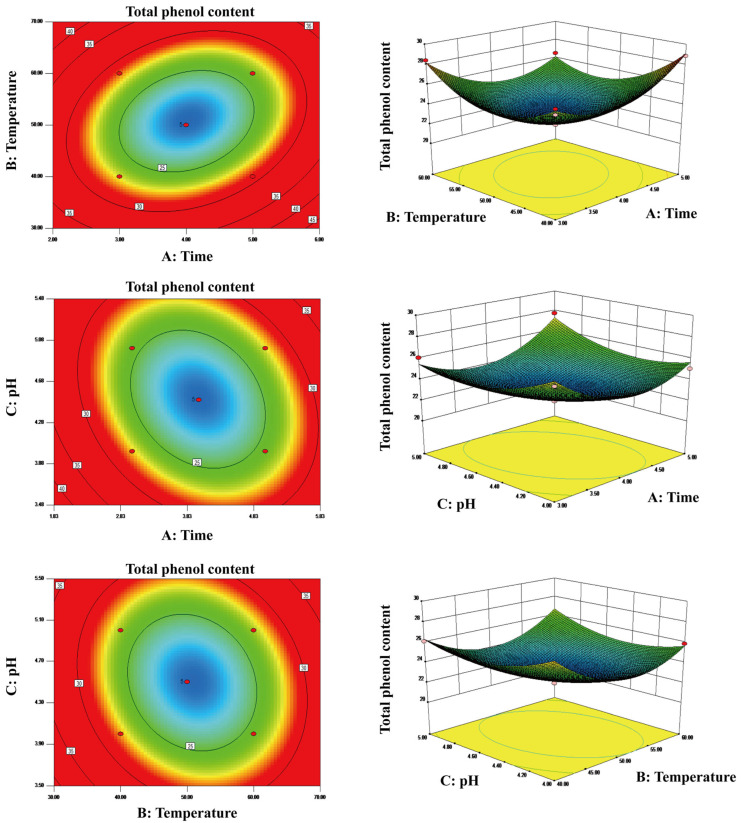
Response surface optimization results of enzymatic digestion parameters in sea buckthorn.

Upon the preliminary findings from single-factor tests, we refined our investigation through a three-factor and three-level response surface experiment. The enzymolysis time (A), temperature (B), and pH (C) were selected as the factors, with total phenolic content serving as the response value (Table S2). The response surface experimental design and results were presented in Table S3. As illustrated in Figure 2 and detailed in Table S4, the optimal enzymatic process parameters for sea buckthorn were determined to be an enzymatic hydrolysis time of 4.01 h, a temperature of 50.77 °C, and a pH of 4.50, resulting in a total phenol content of 22.70 mg/g. Considering the feasibility of the experiments and the convenience of industrial-scale operations, the enzymatic process conditions of sea buckthorn were modified to an enzymatic hydrolysis time of 4.00 h, a temperature of 50.00 °C, and a pH of 4.50. Under such experimental conditions, the total phenol content was 22.48 mg/g, which was close to its theoretical value.

Studies have reported that the optimal pH for debittering cashew juice using pectinase/tannase is 4.61, whereas the highest tannase activity for debittering hickory nuts is achieved at pH 4.0. The pH of 4.50 selected in this study falls within this favorable range, which is beneficial for the synergistic action of both enzymes. Regarding temperature, the optimal temperature determined in this study is 50 °C. In comparison, the optimal temperature for tannase-assisted debittering of hickory nuts is 40 °C, and that for pectinase/tannase-assisted debittering of cashew juice is 35 °C [38,41]. These differences may be attributed to the substrate characteristics. Sea buckthorn pulp is rich in lipids and pectin, and a higher temperature facilitates cell wall softening and pectin dissolution, thereby improving enzyme accessibility. This is consistent with a previous study on pectinase treatment of sea buckthorn, where the optimal temperature was 60 °C [33]. Regarding hydrolysis time, the optimal time of 4 h in this study is longer than the 3 h reported for pectinase-treated sea buckthorn. This is likely because the lower temperature employed in the combined enzyme treatment of sea buckthorn requires a longer duration to promote the release and degradation of phenolic compounds.

### 3.3. Electronic Tongue and Sensory Analysis

**Figure 3:**
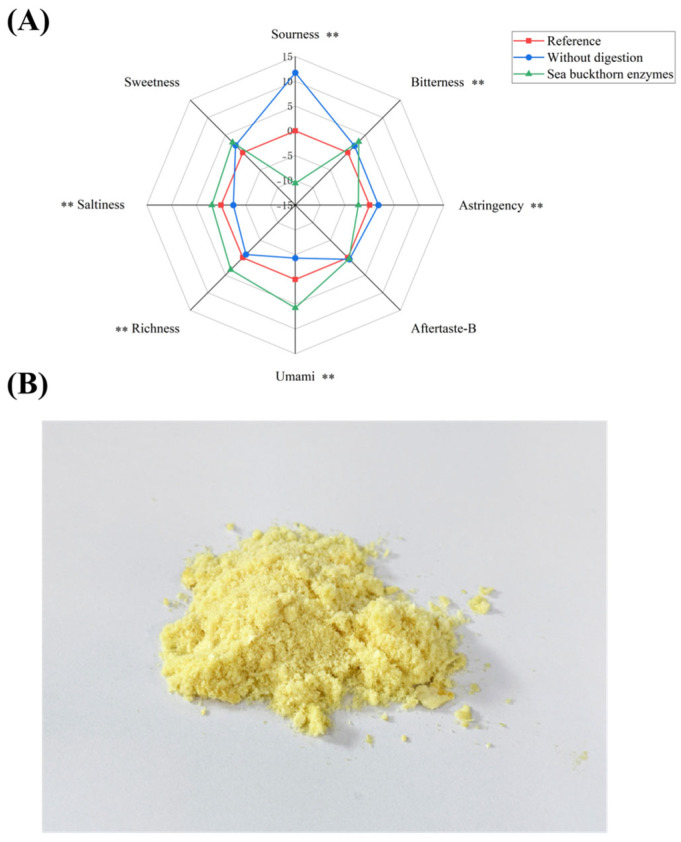
Sea buckthorn flavor analysis results. (**A**) Electronic tongue chart. ** *p* < 0.01 indicated that the enzymolysis group was significantly different compared with the unenzymolysis group. (**B**) Sea buckthorn lyophilized powder post-enzymatic hydrolysis.

Insent’s Taste Analysis System software was utilized to analyze the experimental data from the electronic tongue’s sensors. The resulting six taste attribute values—sourness, sweetness, bitterness, astringency, saltiness, and freshness—are depicted in Figure 3A. Specifically, “Richness” corresponds to the fullness of taste, while “Aftertaste-B” indicates the persistence of bitterness. It was observed that the enzymatic digestion of sea buckthorn resulted in a significant enhancement of sweetness and mouthfeel richness, a marked reduction in sourness and astringency. The bitterness as well as bitter aftertaste were largely comparable to those without enzymatic hydrolysis. Notably, the sourness value of sea buckthorn enzymatic hydrolysate decreased from 11.72 to −10.60, approaching the theoretical sourness detection threshold (−13.00) [42]. This suggested that the hydrolyzed sample retained a very slightly sour taste.

The lyophilized sea buckthorn fruit powder post-enzymatic hydrolysis displayed a pale yellow hue, a bright and lustrous appearance, and demonstrated quick dissolution upon rehydration (Figure 3B). The brew contained no impurities and yielded a clear, transparent juice with characteristics of mild acidity, astringency, and a subtle sweetness. In the sensory evaluation, the lyophilized sea buckthorn fruit powder post-enzymatic digestion demonstrated a significant reduction in acidity and astringency compared to the untreated powder. Its sweetness was notably enhanced, and the characteristic sea buckthorn fragrance was pronounced. The sensory evaluation score was 82.51 ± 0.42, which was consistent with the results obtained by the electronic tongue. Collectively, these results indicated that the enzymatic hydrolysis process effectively refined the taste profile of sea buckthorn lyophilized powder.

Fruit powder is a granular dried material, but it is also inherently loose and exhibits strong hygroscopicity [43]. Internal factors, including moisture, sugars, oils, and fats, as well as external factors such as humidity, temperature, and storage duration, are considered the primary contributors to the caking of fruit and vegetable powders [44]. In prior experiments, sea buckthorn underwent cold pressing, centrifugal oil removal, and vacuum freeze-drying. The resultant enzyme-digested sea buckthorn freeze-dried powder exhibited minimal caking under refrigerated storage and maintained good flowability, which was important in transportation, formulation and mixing, compression and packaging [45].

### 3.4. In Vitro Antioxidant Activity Evaluation of Sea Buckthorn

**Figure 4:**
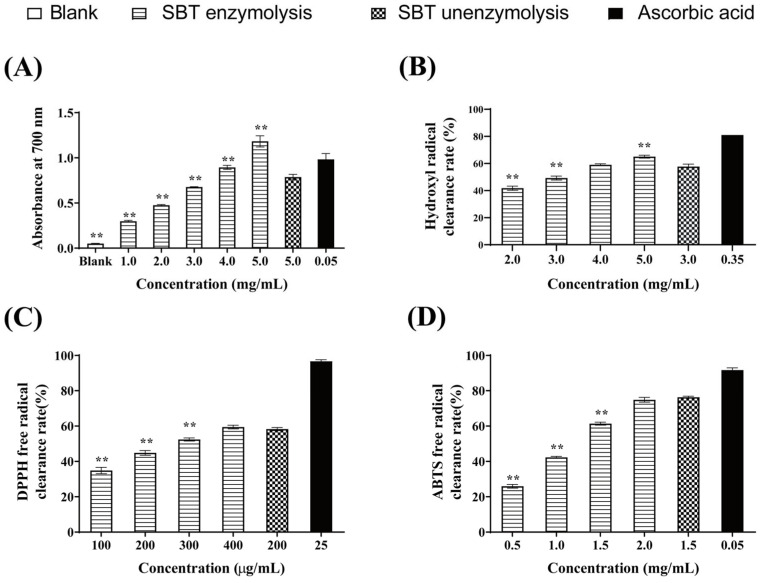
In vitro antioxidant activity assays of sea buckthorn post-enzymatic hydrolysis. (**A**) Total reducing capacity assay. (**B**) Hydroxyl radical scavenging capacity assay. (**C**) DPPH radical scavenging capacity assay. (**D**) ABTS radical scavenging capacity assay. ** *p* < 0.01 indicated that the sample group was significantly different compared with the unenzymolysis group.

The experimental results above demonstrated that enzymatic digestion can effectively improve the sensory flavor of sea buckthorn. However, whether this treatment would have an impact on the antioxidant activity of sea buckthorn remains to be evaluated. As shown in Figure 4, all of the four antioxidant activity assays exhibited a positive correlation between the sample concentrations and antioxidant capacity of sea buckthorn after enzymatic hydrolysis treatment. Notably, at a concentration of 4.0 mg/mL, the enzymatic hydrolysate of sea buckthorn demonstrated a higher total reducing power compared to the unenzymolysis group at 5.0 mg/mL, and was nearly equivalent to that of 0.05 mg/mL ascorbic acid (Figure 4A). The hydroxyl radical scavenging activity of the hydrolysate at 4 mg/mL matched that of the unenzymolysis group at 3.0 mg/mL, and the 5.0 mg/mL sample could reach a 65.0% hydroxyl radical scavenging rate (Figure 4B). The DPPH radical clearance experiment showed that the hydrolysate of sea buckthorn had an IC50 of 276.7 μg/mL and at 400.0 μg/mL, it exhibited equivalent antioxidant capacity to 200 μg/mL sea buckthorn lyophilized powder without digestion (Figure 4C). For ABTS radical scavenging ability, the hydrolysate at 2.0 mg/mL was comparable to the lyophilized powder at 1.5 mg/mL (Figure 4D). In short, the enzymatic hydrolysate of sea buckthorn still maintained considerable antioxidant activity in vitro, indicating that composite enzymatic hydrolysis did not severely impair its functional properties.

Sea buckthorn is naturally rich in antioxidant compounds. The concentration-dependent antioxidant activities observed in this study suggest that moderate enzymatic conditions (50 °C and pH 4.5) may have minimized excessive degradation of thermolabile compounds. In addition, pectinase-mediated cell wall degradation could facilitate the release of antioxidant substances [33,46]. Research on sea buckthorn leaves revealed that the polysaccharide content after enzymatic hydrolysis by pectinase increased from 84.51 to 224.93 mg/g, and exhibited stronger scavenging ability against DPPH and hydroxyl radicals compared to non-hydrolyzed counterparts [24]. Meanwhile, tannase hydrolysis can reduce astringency by degrading tannins but may simultaneously transform high-molecular-weight polyphenols into smaller antioxidant-active molecules, thereby partially preserving antioxidant capacity. The enzymatic hydrolysate exhibited slightly lower antioxidant performance than the untreated sample at equivalent concentrations in some assays, likely due to partial transformation of some bioactive substances during hydrolysis.

### 3.5. Protective Effect of Sea Buckthorn Against Oxidative Stress in Cells

**Figure 5:**
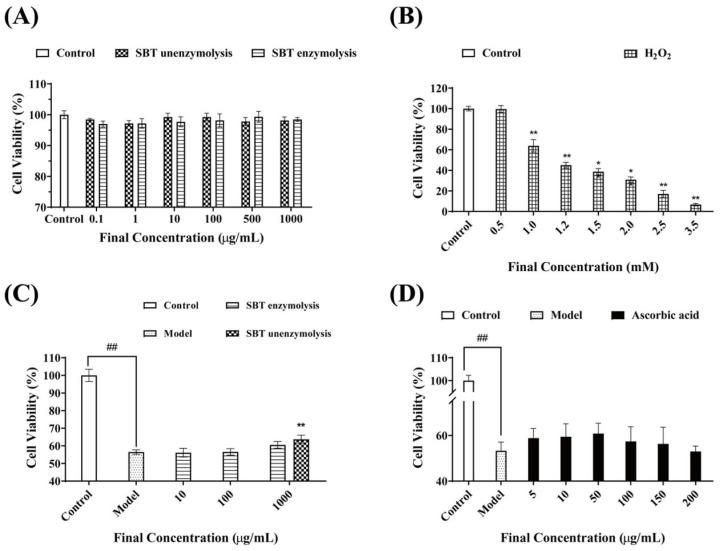
Cellular protection effect against oxidative stress. (**A**) Safety evaluation of sea buckthorn on LO2 cells before and after enzyme digestion. (**B**) Effects of H_2_O_2_ on the survival of LO2 cells. (**C**) Protective effects of sea buckthorn post-enzymatic hydrolysis on LO2 cells damaged by H_2_O_2_. (**D**) Protective effects of ascorbic acid on oxidatively damaged cells. In (**B**), * *p* < 0.05, ** *p* < 0.01 indicated that the sample group was significantly different compared with the control group. In (**C**,**D**), ## *p* < 0.01 indicated that the difference between the model group and the control group was significant; ** *p* < 0.01 indicated that the difference between the sample group and the model group was significant.

When the levels of reactive oxygen species (ROS) and reactive nitrogen species (RNS) in the body are excessively elevated, the antioxidant system becomes imbalanced, resulting in oxidative stress. This condition induces damage to organs and tissues, triggers inflammatory responses, and poses significant health risks. LO2 cell oxidative injury model has been widely utilized to assess the antioxidant activity of test substances. As shown in Figure 5A, sea buckthorn before and after enzymatic hydrolysis had no cytotoxicity on LO2 cells over the range of 0.1~1000 μg/mL. H_2_O_2_ incubation at a range of 1.0~3.5 mM obviously caused cell damage, leading to a significant decline in LO2 survival rate (Figure 5B). Since a semi-lethal cell rate was observed when the H_2_O_2_ concentration was near 1.2 mM, this concentration was used to construct an oxidative stress model in LO2 cells. After incubating the sea buckthorn hydrolysate with damaged cells, we found that compared with the model group, the sea buckthorn hydrolysate at concentrations of both 10 μg/mL and 100 μg/mL did not exhibit a significant protective effect. However, when the tested concentration reached 1000 μg/mL, the survival rate notably increased to 60.56 ± 2.0% and was close to that of the unenzymolysis group at the same concentration (Figure 5C). A similar phenomenon was observed in grape pomace treated with tannase. The enzymatically hydrolyzed and non-hydrolyzed grape pomace did not show a dose-dependent effect, and their antioxidant capacities at the cellular level were comparable [47]. Despite this, it was also shown that their protective efficacy was similar to the maximum protective effect of the ascorbic acid group in this study (50 μg/mL) (Figure 5D). Collectively, these results demonstrated that sea buckthorn post-enzymatic hydrolysis retained a certain protective effect on LO2 cells exposed to H_2_O_2_.

### 3.6. Metabolomic Analysis of Sea Buckthorn Post-Enzymatic Hydrolysis

#### 3.6.1. Evaluation of Experimental Data Quality

**Figure 6:**
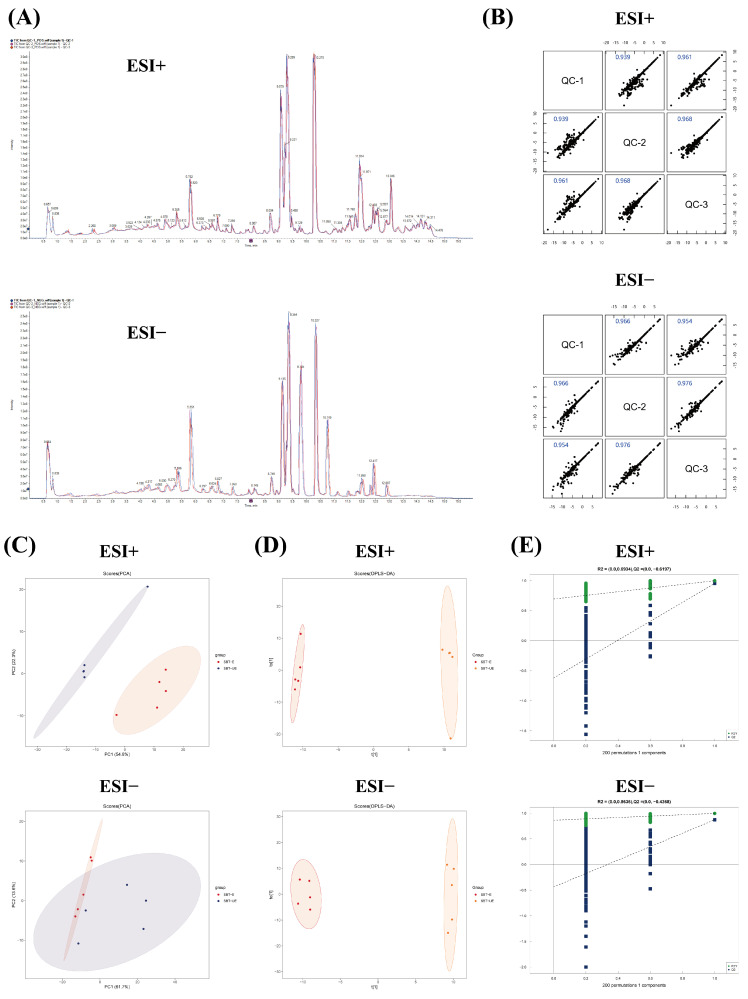
Metabolomics data analysis and quality assessment. (**A**) TIC plot. (**B**) QC sample correlation plot. (**C**) PCA score chart. (**D**) OPLS-DA score chart. (**E**) OPLS-DA permutation test diagram.

The total ion current (TIC) plots of the quality control (QC) samples collected at ESI+ and ESI- are shown in Figure 6A. The peak areas and retention times were consistent across all QC samples, indicating high reproducibility. The correlation coefficients for QC sample replicates in both modes exceeded 0.9, which signified minimal experimental errors and excellent data quality (Figure 6B). Through principal component analysis (PCA) of SBT-E (SBT enzymolysis) and SBT-UE (SBT unenzymolysis), the samples from both groups were found to cluster within a relatively small area with partial overlap. This indicated that there was a certain gap between the two groups, yet the metabolic differences were minor, suggesting that enzymatic hydrolysis induced metabolic changes in sea buckthorn (Figure 6C). An orthogonal partial least squares discriminant analysis (OPLS-DA) was used to highlight the variation in metabolites between the two groups. As Figure 6D shown, the OPLS-DA model emphasized the maximum inter-group differences along the horizontal coordinate t [1], with SBT-E located to the left of the confidence interval. All evaluation parameters for the model exceeded the threshold of 0.5, indicating the model’s stability and reliability. The cross-validation with 200 permutation tests showed that both R2 and Q2 of the model progressively declined as the permutation retention decreased, and the R2 and Q2 on the rightmost side were always higher than those on the left. That meant the OPLS-DA model did not exhibit overfitting, further validating its reliability. (Figure 6E). Overall, the enzyme digestion reaction indeed produced a significant effect on the metabolites in sea buckthorn.

#### 3.6.2. Identification of Differential Metabolites in Sea Buckthorn Before and After Enzymatic Hydrolysis

**Figure 7:**
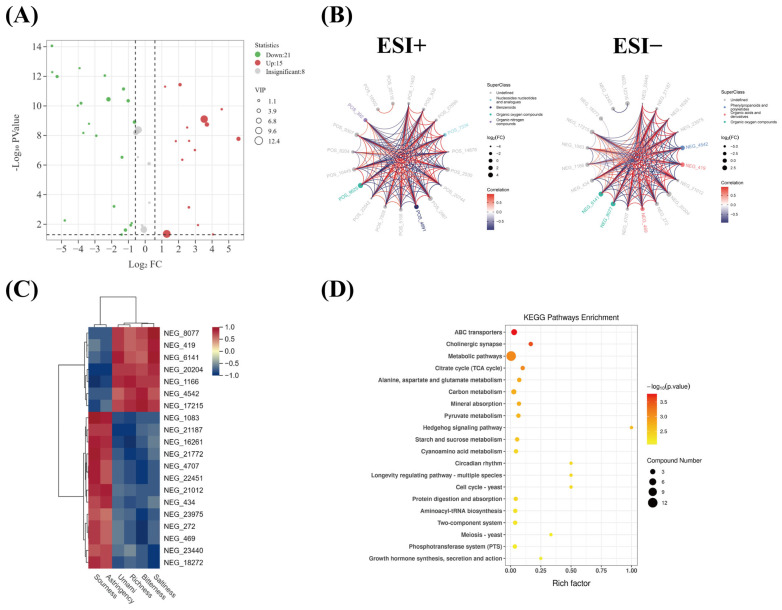
Identification of differential metabolites and relevance analysis. (**A**) Volcanic map of different metabolites of sea buckthorn before and after enzymatic hydrolysis. (**B**) Correlation analysis and chord charts. (**C**) Correlation of differential metabolites of sea buckthorn before and after enzymatic hydrolysis with sensory evaluation of e-tongue (correlation coefficient |r| > 0.8, *p* < 0.05). (**D**) KEGG enrichment pathway diagram. The size of the bubble represented the “rich factor” in the pathway, and the bubble color represented the enrichment value corresponding to each pathway. The value increased from small to large and the color changed from yellow to red.

In both positive and negative ion modes, 36 significant differential metabolites between SBT-E and SBT-UE were identified through a comparative analysis with the KEGG compound database. These metabolites were screened based on a fold change (FC) greater than 1.5 or less than 0.67, and a *p*-value less than 0.05 (Figure 7A and Table S5). Among these, 15 metabolites were significantly upregulated, including fructose, alpha-D-glucopyranoside methyl, trehalose, beta-gentiobiose, quercetin, sucrose, ginsenoside Rh1, isorhamnetin 3-galactoside, etc. Conversely, 21 metabolites were significantly downregulated, encompassing qinpiroside, fraxin, lutein, vitamin E, fumaric acid, L-malic acid, xanthorhamnin, ascorbic acid, etc.

The plant cell wall is an intricate network of polysaccharides (pectin and cellulose), proteins and aromatic polymers. The reason for the significant changes in glycoconjugates and aromatic compounds during enzymatic digestion may be that the pectinase catalyzes glycosidic bonds within plant cell walls, degrading pectin and producing functional low molecular weight sugars. In sea buckthorn, metabolites such as organic acids, sugars, polyphenols and amino acids are not only responsible for the actual growth, development and physiological function status of the tree, but are also directly related to the quality, flavor and taste of sea buckthorn fruit. Key sugars such as sucrose and fructose contribute to the sweetness of sea buckthorn fruits, while its pronounced sourness, astringency, and bitterness arise from its high malic acid content [19]. Modulating the contents of sugars and malic acid is key to improving the taste of sea buckthorn juice. For example, Wang et al. reported that after pectinase treatment and subsequent fermentation with *Saccharomyces cerevisiae*, the malic acid content in sea buckthorn juice decreased from 24.06 g/L to 8.78 g/L, total phenolic content decreased by 4.22% compared with the control group, and the sour and astringent off-flavors were significantly alleviated [25]. In this study, saccharides increased significantly after enzymatic hydrolysis, whereas the contents of organic acids such as malic acid decreased markedly, indicating that pectinase/tannase enzymatic digestion is an effective strategy for improving the sensory quality and other properties of sea buckthorn lyophilized powder.

In fruit deastringency treatments, a reduction in soluble tannin content is typically accompanied by a decrease in total phenolic content and the corresponding antioxidant activity [20]. L-Malic acid, ascorbic acid, fumaric acid, and vitamin E are important antioxidant constituents in sea buckthorn. A significant reduction in their content may primarily account for the diminished antioxidant capacity following enzymatic hydrolysis. Changes in antioxidant properties are associated with the chemical composition of the substrates, interactions among other components, and the conditions of enzymatic hydrolysis. Heat-sensitive or easily oxidized constituents, such as certain vitamins (e.g., vitamin C), may undergo degradation as a function of treatment intensity and duration. However, metabolomics also showed significant upregulation of phenolic antioxidants such as quercetin and isorhamnetin-3-O-galactoside, which may partially compensate for the functional loss. Consequently, the freeze-dried sea buckthorn powder retained considerable antioxidant activity even after complex enzymatic hydrolysis aimed at improving its sensory properties.

#### 3.6.3. Relevance Analysis

Figure 7B showed the chord diagrams of significantly different metabolites in sea buckthorn before and after enzymatic digestion, with a correlation coefficient |r| > 0.8 and *p* < 0.05. In our analysis, alginose (POS_9625) from organic oxygen compounds and choline (POS_392) from organic nitrogen compounds displayed a robust positive correlation (r = 0.97). Sucrose (NEG_6141) and lactose (NEG_8077), part of the organic oxygen compounds, showed an exceptionally strong positive linkage (r = 0.99). Quercetin (NEG_4542) from the phenylacetone and polyketide compounds group also correlated positively with both sucrose and lactose (r = 0.99). Interestingly, within the organic acids and derivatives group, L-malic acid (NEG_469) and isoleucine group (NEG_419) showed a pronounced negative correlation (r = −0.99). It was noteworthy that both L-malic acid (NEG_469) from the organic acid group and cyclic adenosine (POS_7334) from the nucleotide group showed significant negative correlations with other metabolites, suggesting their potential role in the metabolic synthesis of other compounds and highlighting their importance in anabolic pathways. During the enzymatic hydrolysis of sea buckthorn, oxygen-containing organic compounds showed positive correlations with nitrogen-containing organic compounds, as well as with phenylacetone and polyketones, suggesting that the significant metabolites may originate from the same biosynthetic pathway.

Figure 7C illustrates the correlation between the differential metabolites resulting from enzymatic hydrolysis of sea buckthorn and the sensory attributes as evaluated by an electronic tongue. Lactose (NEG_8077), L-isoleucine (NEG_419), sucrose (NEG_6141), ginsenoside Rh1 (NEG_20204), D-glucose (NEG_1166), quercetin (NEG_4542), and kaempferol-3-O-glucoside-6’’-p-coumaroyl (NEG_17215) exhibited significant negative correlations with sourness and astringency, while showing significant positive correlations with saltiness, richness, and bitterness. Conversely, ascorbic acid (NEG_1083), PG 32:1 (NEG_21187), (2-hydroxy-3-octadec-9-enoyloxypropyl) 2-(trimethylazaniumyl)ethyl phosphate (NEG_16261), PE 36:2 (NEG_21772), xanthorhamnin (NEG_22451), L-glutathione (NEG_4707), PE 34:3 (NEG_21012), asparagine (NEG_434), PI 34:2 (NEG_23975), fumaric acid (NEG_272), L-malic acid (NEG_469), PI 32:1 (NEG_23440), and isorhamnetin-3-O-galactoside-6’’-rhamnoside (NEG_18272) demonstrated significant positive correlations with sourness and astringency, and significant negative correlations with savory taste, richness, bitterness, and umami. These results suggested that changes in the content of organic acids and derivatives may be responsible for changes in sourness, while shifts in phenolic metabolite content might be driving the changes in bitter and astringent taste perceptions. Alterations in lipids and their molecular constituents, carbohydrate metabolites, and amino acid derivatives may be influencing the richness and saltiness of the taste profiles. This was in line with the findings of Adam et al. [48], who had shown that phenolic acids and other polyphenolic compounds were the key active substances affecting astringency as well as antioxidant activity of fruits.

Figure 7D presented the KEGG pathway enrichment analysis, which revealed that the metabolic differences in sea buckthorn before and after enzymatic hydrolysis were primarily focused on these pathways: ABC transporters, cholinergic synapse, metabolic pathways, citrate cycle, as well as alanine, aspartate and glutamate metabolism. In general, sweetness and acidity are the most important taste sensory evaluations in fruits, while the concentration and composition of sugars and organic acids are important determinants of the quality of fruits. ABC transporters showed the most pronounced metabolic differences in sea buckthorn before and after enzymatic digestion, and metabolic pathways were identified as the most significantly enriched. Thereby, we hypothesized that ABC transporters and metabolic pathways were likely the two most critical metabolic pathways influencing the sensory taste, nutritional content, and aroma profile of sea buckthorn.

## 4. Conclusions

In this research, we identified the optimal conditions for the enzymatic hydrolysis of sea buckthorn fruit through single-factor experiments and response surface methodology experiments. The ideal parameters were found to be an enzymatic pH of 4.50, a composite enzyme ratio of pectinase to tannase at 1:1 (weight/weight), an enzyme dosage of 0.20%, a temperature of 50 °C, and a duration of 4 h. The resulting lyophilized sea buckthorn fruit powder exhibited a bright color and luster, a significant reduction in sourness and astringency, high solubility, absence of impurities post-brewing, and a distinctive aroma. The enzymatic processing maintained the antioxidant potential of sea buckthorn fruit. But it should be noted that this study employed a single-parameter optimization based solely on total phenolic content, which may oversimplify the trade-off between reducing astringency/astringency and preserving antioxidant capacity. Future studies should incorporate multi-response optimization including sensory evaluation and antioxidant assays to validate the findings.

In metabolomic analysis, a total of 36 differential metabolites were identified before and after enzymatic hydrolysis, encompassing alkaloids and their derivatives, aromatic compounds, hydrocarbon derivatives, lignans and related compounds, lipids and lipid-like molecules. Changes in the levels of organic acids, phenolics, and carbohydrates were primarily responsible for the altered taste profiles. Moreover, the ABC transporter and metabolic pathways were identified as potentially the most influential routes modulating the sensory and nutritional properties of sea buckthorn during enzymatic processing.

In summary, the sea buckthorn fruit powder post-enzymatic hydrolysis had superior taste qualities along with satisfying antioxidant efficacy. Untargeted metabolomics revealed the changes in metabolites during the enzymatic hydrolysis process, providing a theoretical basis for understanding the physiological and biochemical functions of sea buckthorn.

## Data Availability

The original contributions presented in this study are included in the article and Supplementary Materials. Further inquiries can be directed to the corresponding authors.

## Supplementary Materials

The following supporting information can be downloaded at: https://www.mdpi.com/article/10.3390/foods15122240/s1, Table S1: Sensory score standard; Table S2: Response surface experiment factors and levels; Table S3: Response surface experimental design program and results; Table S4: Comparison of theoretical values for response surface optimization and actual values for enzymatic hydrolysis of sea buckthorn; Table S5: Significantly different metabolites before and after enzymatic hydrolysis of sea buckthorn.

## References

[1] Zheng W.-H., Bai H.-Y., Han S., Bao F., Zhang K.-X., Sun L.-L., Du H., Yang Z.-G. (2019). Analysis on the Constituents of Branches, Berries, and Leaves of *Hippophae rhamnoides* L. by UHPLC-ESI-QTOF-MS and Their Anti-Inflammatory Activities. Nat. Prod. Commun..

[2] Zhang Z., Chen Y., Chen Z., Gao Z., Cheng Y., Qu K. (2024). Quality Analysis and Assessment of Representative Sea Buckthorn Fruits in Northern China. Food Chem. X.

[3] Mei D., Ma X., Fu F., Cao F. (2023). Research Status and Development Prospects of Sea buckthorn (*Hippophae rhamnoides* L.) Resources in China. Forests.

[4] Guo R., Guo X., Li T., Fu X., Liu R.H. (2017). Comparative Assessment of Phytochemical Profiles, Antioxidant and Antiproliferative Activities of Sea Buckthorn (*Hippophaë rhamnoides* L.) Berries. Food Chem..

[5] Jubayer M.F., Mazumder M.A.R., Nayik G.A., Ansari M.J., Ranganathan T.V., Sharma A., Nayik G.A. (2023). *Hippophae rhamnoides* L.: Sea Buckthorn. Immunity Boosting Medicinal Plants of the Western Himalayas.

[6] Wang K., Xu Z., Liao X. (2022). Bioactive Compounds, Health Benefits and Functional Food Products of Sea Buckthorn: A Review. Crit. Rev. Food Sci. Nutr..

[7] Yu W., Du Y., Li S., Wu L., Guo X., Qin W., Kuang X., Gao X., Wang Q., Kuang H. (2024). Sea Buckthorn-nutritional Composition, Bioactivity, Safety, and Applications: A Review. J. Food Compos. Anal..

[8] Zhu P., Ren Y., Wei C., Luo J., Wu D., Ye X., Donlao N., Tian J. (2025). Compounds from Sea Buckthorn and Their Application in Food: A Review. Food Chem..

[9] Ciesarova Z., Murkovic M., Cejpek K., Kreps F., Tobolkova B., Koplik R., Belajova E., Kukurova K., Dasko L., Panovska Z. (2020). Why is Sea Buckthorn (*Hippophae rhamnoides* L.) So Exceptional? A review. Food Res. Int..

[10] Attri S., Goel G. (2018). Influence of Polyphenol Rich Seabuckthorn Berries Juice on Release of Polyphenols and Colonic Microbiota on Exposure to Simulated Human Digestion Model. Food Res. Int..

[11] Ji M., Gong X., Li X., Wang C., Li M. (2020). Advanced Research on the Antioxidant Activity and Mechanism of Polyphenols from Hippophae Species-A Review. Molecules.

[12] Suchal K., Bhatia J., Malik S., Malhotra R.K., Gamad N., Goyal S., Nag T.C., Arya D.S., Ojha S. (2016). Seabuckthorn Pulp Oil Protects against Myocardial Ischemia-Reperfusion Injury in Rats through Activation of Akt/eNOS. Front. Pharmacol..

[13] Wang H., Bi H., Gao T., Zhao B., Ni W., Liu J. (2018). A Homogalacturonan from *Hippophae rhamnoides* L. Berries Enhance Immunomodulatory Activity through TLR4/MyD88 Pathway Mediated Activation of Macrophages. Int. J. Biol. Macromol..

[14] Yan Z., Feng X., Li X., Gao Z., Wang Z., Ren G., Long F. (2024). Sea Buckthorn Flavonoid Extracted by High Hydrostatic Pressure Inhibited IgE-Stimulated Mast Cell Activation through the Mitogen-Activated Protein Kinase Signaling Pathway. Foods.

[15] Piłat B., Bieniek A., Zadernowski R. (2015). Common Sea Buckthorn (*Hippophae rhamnoides* L.) as An Alternative Orchard Plant. Pol. J. Nat. Sci..

[16] Wang Z., Zhao F., Wei P., Chai X., Hou G., Meng Q. (2022). Phytochemistry, Health Benefits, and Food Applications of Sea Buckthorn (*Hippophae rhamnoides* L.): A Comprehensive Review. Front. Nutr..

[17] Ma X., Laaksonen O., Zheng J., Yang W., Trépanier M., Kallio H., Yang B. (2016). Flavonol Glycosides in Berries of Two Major Subspecies of Sea Buckthorn (*Hippophaë rhamnoides* L.) and Influence of Growth Sites. Food Chem..

[18] Ma X., Yang W., Marsol-Vall A., Laaksonen O., Yang B. (2020). Analysis of Flavour Compounds and Prediction of Sensory Properties in Sea Buckthorn (*Hippophaë rhamnoides* L.) Berries. Int. J. Food Sci. Technol..

[19] Ma X., Yang W., Laaksonen O., Nylander M., Kallio H., Yang B. (2017). Role of Flavonols and Proanthocyanidins in the Sensory Quality of Sea Buckthorn (*Hippophaë rhamnoides* L.) Berries. J. Agric. Food Chem..

[20] Zhao W., Zheng M., Li X., Song K., Shi D. (2025). Fruit Astringency: Mechanisms, Technologies, and Future Directions. Horticulturae.

[21] Oladokun O., Tarrega A., James S., Smart K., Hort J., Cook D. (2016). The Impact of Hop Bitter Acid and Polyphenol Profiles on the Perceived Bitterness of Beer. Food Chem..

[22] Wang Y., Gong S., Chen J., Chen G., Feng Z., Yin J. (2026). Mechanisms of Astringency Modulation: How Sugars and Organic Acids Modulate Epigallocatechin Gallate Astringency in New-style Fruit Tea. Food Res. Int..

[23] Ninga K.A., Sengupta S., Jain A., Desobgo Z.S.C., Nso E.J., De S. (2018). Kinetics of Enzymatic Hydrolysis of Pectinaceous Matter in Guava Juice. J. Food Eng..

[24] An Y., Wang B., Meng Z., Song Y., Wang Y., Wang W., Xu M., An X. (2024). Optimization of the Enzymatic Hydrolysis Process for Sea Buckthorn Leaf Polysaccharides: An Investigation into Their Enhanced Physicochemical Properties and Antioxidant Activities. Chem. Biol. Technol. Agric..

[25] Wang J., Zhang Y., Zhang B., Han Y., Li J., Zhang B., Jiang Y. (2025). Optimization of the Quality of Sea Buckthorn Juice by Enzymatic Digestion and Inoculation Sequence. Food Chem..

[26] Sayed Mostafa H. (2023). Production of Low-tannin Hibiscus Sabdariffa Tea through D-optimal Design Optimization of the Preparation Conditions and the Catalytic Action of New Tannase. Food Chem. X.

[27] Geetha P.S. (2020). Storage studies of enzyme clarified astringency free cashew apple juice and its value-added products. J. Pharmacogn. Phytochem..

[28] Ding Y., Bi J., Chen J., Chen Q., Morozova K., Scampicchio M., Zhou M. (2024). The Occurring of Astringency during Persimmon Pulp Drying and Its Correlation with Tannin Derivatives. J. Food Compos. Anal..

[29] Lu H., Hu Y., Han B., Li Z., Guo Q. (2025). Optimization of Preparation Process of Anthocyanin-rich NFC Blueberryjuice. Cereal. Oils.

[30] Matić P., Sabljić M., Jakobek L. (2017). Validation of Spectrophotometric Methods for the Determination of Total Polyphenol and Total Flavonoid Content. J. AOAC Int..

[31] Box G.E.P., Behnken D.W. (1960). Some New Three Level Designs for the Study of Quantitative Variables. Technometrics.

[32] Yang H., Cai G., Lu J., Gómez Plaza E. (2021). The Production and Application of Enzymes Related to the Quality of Fruit Wine. Crit. Rev. Food Sci. Nutr..

[33] Guo K. (2024). Changes in the Main Physicochemical Properties and Electrochemical Fingerprints in the Production of Sea Buckthorn Juice by Pectinase Treatment. Molecules.

[34] Li Y.-C., Fu Y.-Q., Gao Y., Wang J.-Q., Jin S., Liu Y., Chen J.-X., Xu Y.-Q. (2025). Enzyme-assisted Dynamic Extraction as A Promising Method to Produce High-quality Fresh Tea Juice from Summer Tea Leaves. Food Chem..

[35] Xiang H., Waterhouse D.-S., Liu P., Waterhouse G.I.N., Li J., Cui C. (2020). Pancreatic Lipase-inhibiting Protein Hydrolysate and Peptides from Seabuckthorn Seed Meal: Preparation Optimization and Inhibitory Mechanism. LWT.

[36] Reynolds A.G., Knox A., Di Profio F. (2018). Evaluation of Macerating Pectinase Enzyme Activity under Various Temperature, pH and Ethanol Regimes. Beverages.

[37] Ozojiofor U., Rasheed Z.A. (2024). Pectinases: Structure, Functions and Biotechnological Applications. J. Appl. Nat. Sci..

[38] Hanh N., Trang N., Anh N., Huong N., Hưng N., Trang V. (2023). Removal of Tannins from Cashew (*Anacardium occidentale* L.) Apple Juice in Binh Phuoc (Viet Nam) by Using Enzymatic Method. J. Law Sustain. Dev..

[39] Yang B., Linko A.-M., Adlercreutz H., Kallio H. (2006). Secoisolariciresinol and Matairesinol of Sea Buckthorn (*Hippophaë rhamnoides* L.) Berries of Different Subspecies and Harvesting Times. J. Agric. Food Chem..

[40] Zhu Y., Ji X., Yuen M., Yuen T., Yuen H., Wang M., Smith D., Peng Q. (2022). Effects of Ball Milling Combined With Cellulase Treatment on Physicochemical Properties and in vitro Hypoglycemic Ability of Sea Buckthorn Seed Meal Insoluble Dietary Fiber. Front. Nutr..

[41] He T., Wang C., Wang M., Wang K., Huang C., Guo W., Shi D., Hu H., Wu Y., Wang J. (2025). Elucidation of the Potential Mechanism of Tannase in Removing the Astringency of Hickory Nuts and Its Effect on Flavor Profile Utilizing Wide-Targeted Metabolomics, E-nose, and HS-SPME-GC–MS. Food Res. Int..

[42] Gao S., Hu Y.-Y., Qian W.-Z., Xiao Y.-X., Liao J.-L., Chen J.-C. (2026). Sourness Signature during Prunus Mume Fruit Development: Integrative Insight from Metabolomics, Organic Acid Profiles, E-tongue, and Computational Binding Simulation. Food Chem..

[43] Saifullah M., Yusof Y.A., Chin N.L., Aziz M.G. (2016). Physicochemical and Flow Properties of Fruit Powder and Their Effect on the Dissolution of Fast Dissolving Fruit Powder Tablets. Powder Technol..

[44] Karam M.C., Petit J., Zimmer D., Baudelaire Djantou E., Scher J. (2016). Effects of Drying and Grinding in Production of Fruit and Vegetable Powders: A Review. J. Food Eng..

[45] Knowlton T., Klinzing G., Yang W.C., Carson J.W. (1994). The Importance of Storage, Transfer, and Collection. Chem. Eng. Prog..

[46] Rösch D., Bergmann M., Knorr D., Kroh L.W. (2003). Structure–Antioxidant Efficiency Relationships of Phenolic Compounds and Their Contribution to the Antioxidant Activity of Sea Buckthorn Juice. J. Agric. Food Chem..

[47] Martins I.M., Macedo G.A., Macedo J.A., Roberto B.S., Chen Q., Blumberg J.B., Chen C.-Y.O. (2017). Tannase Enhances the Anti-inflammatory Effect of Grape Pomace in Caco-2 Cells Treated with IL-1β. J. Funct. Foods.

[48] Drewnowski A., Gomez-Carneros C. (2000). Bitter taste, Phytonutrients, and the Consumer: A Review. Am. J. Clin. Nutr..

