# Supplementary material for: Enzymatic Deastringent Fruit Powder of Sea Buckthorn (Hippophae rhamnoides L.): Preparation, Antioxidant Activity Investigation and Metabolomics Analysis
Source: Foods. 2026 Jun 21;15(12):2240. doi: 10.3390/foods15122240 (PMC13298325; doi:10.3390/foods15122240)
Supplement: Supplementary file 1 [file foods-15-02240-s001.zip › foods-4342590-supplementary.pdf]

Table S1

Sensory score standard

Evaluation indicators	Sensory Scoring Criteria	Points (100-point scale)
Appearance Colour (20%)	The powder is dry and shiny, yellow in colour	15~20
	The powder is dry and brownish in colour	8~14
	Powdery agglomerates without luster, brownish in colour	0~7
Organisational pattern （20%）	Good rehydration, no agglomeration after rehydration, homogeneous and free of impurities	15~20
	Dissolved basically after rehydration, with a small amount of precipitation and impurities	8~14
	Large amounts of sediment and impurities present after rehydration	0~7
Odours （30%）	With the unique aroma of sea buckthorn, no peculiar aroma	21~30
	Sea buckthorn aroma is small, no foreign aroma	11~20
	No aroma or weak aroma, unusual aroma	0~10
Texture （30%）	Slightly acidic taste, soft and non-irritating in the mouth, not obvious astringency	21~30
	More acidic, astringent in the mouth, more irritating in the mouth	11~20
	Sour taste and pronounced irritation	0~10

Table S2

Response surface experiment factors and levels

Factors	Level		
	-1	0	1
A Time（h）	3	4	5
B Temperature（℃）	40	50	60
C pH	4	4.5	5

Table S3

Response surface experimental design programme and results

Test number	Time（h）	Temperature（℃）	pH	Total Phenol Content（mg/g）
1	3	40	4.5	26.44
2	5	40	4.5	28.86
3	3	60	4.5	28.42
4	5	60	4.5	26.53
5	3	50	4	26.79
6	5	50	4	25.06
7	3	50	5	26.09
8	5	50	5	27.73
9	4	40	4	28.3
10	4	60	4	25.89
11	4	40	5	26.12
12	4	60	5	25.91
13	4	50	4.5	22.8
14	4	50	4.5	21.93
15	4	50	4.5	23.56
16	4	50	4.5	22.33
17	4	50	4.5	22.93

Table S4

Comparison of theoretical values for response surface optimisation and actual values for enzymatic hydrolysis of sea buckthorn

	Time（h）	Temperature（℃）	pH	Total Phenol Content（mg/g）
Theoretical value	4.01	50.77	4.50	22.70
Actual value	4	50	4.5	22.48

Table S5

Significantly different metabolites before and after enzymatic hydrolysis of sea buckthorn

ID	Name	Class	Fold change
NEG_23440	PI 32:1		0.020982365
NEG_21187	PG 32:1		0.066109943
NEG_16261	(2-Hydroxy-3-octadec-9-enoyloxypropyl) 2-(trimethylazaniumyl)ethyl phosphate	Glycerophospholipids	0.021273573
NEG_23975	PI 34:2		0.026022559
NEG_4542	Quercetin	Flavonoids	4.24714048
NEG_419	l-Isoleucine	Carboxylic acids and derivatives	2.285036569
NEG_21012	PE 34:3		0.070152661
NEG_20204	Ginsenoside Rh1		23.88233103
NEG_272	Fumaric acid	Carboxylic acids and derivatives	0.643925004
NEG_469	L-Malic acid	Hydroxy acids and derivatives	0.669994397
NEG_4707	L-Glutathione (reduced form)	Carboxylic acids and derivatives	0.132197397
NEG_8077	Lactose		6.175859064
NEG_6141	Sucrose	Organooxygen compounds	7.857042433
NEG_434	Asparagine	Carboxylic acids and derivatives	0.740142175
NEG_1166	D-Glucose	Organooxygen compounds	1.191958783
NEG_1083	Ascorbic acid	Dihydrofurans	0.27489574
NEG_17215	Kaempferol-3-O-glucoside-6"-p-coumaroyl		6.121204049
NEG_21772	PE 36:2		0.0357171
NEG_18272	Isorhamnetin-3-O-galactoside-6"-rhamnoside		0.945667153
NEG_22451	Xanthorhamnin	Flavonoids	0.442087214
NEG_12316	Isorhamnetin 3-galactoside	Flavonoids	2.446966626
NEG_2685	Citrinin	Benzopyrans	16.65335044
POS_11402	Fraxin		0.18808455
POS_938	Trigonelline HCl		0.407891808

Table S5 (continued)

ID	Name	Class	Fold change
POS_27690	(2R,3S,4R,5R,8R,10R,11R,13S,14R)-11-[(2S,3R,4S,6R)-4-(Dimethylamino)-3-hydroxy-6-methyloxan-2-yl]oxy-2-ethyl-3,4,10-trihydroxy-13-[(2R,4R,5S,6S)-5-hydroxy-4-methoxy-4,6-dimethyloxan-2-yl]oxy-3,5,6,8,10,12,14-heptamethyl-1-oxa-6-azacyclopentadecan-15-one	Organooxygen compounds	0.218590182
POS_7334	Cyclic AMP	Purine nucleotides	0.49543252
POS_14678	Tetracycline	Tetracyclines	0.061294345
POS_2530	Fructose (Generic Ketohexose)	Organooxygen compounds	11.5319252
POS_20744	Lutein	Prenol lipids	0.097569362
POS_2987	alpha-D-Glucopyranoside, methyl	Organooxygen compounds	12.91166067
POS_4891	Clenbuterol	Benzene and substituted derivatives	5.706309883
POS_5166	9-Octadecenamide	Fatty Acyls	0.755129717
POS_7828	Erucamide	Fatty Acyls	0.695263753
POS_22542	3-{12-hydroxy-12-[5'-(1-hydroxydodecyl)-[2,2'-bioxolan]-5-yl]dodecyl}-5-methyl-5H-furan-2-one		0.077228109
POS_9625	Trehalose	Organooxygen compounds	47.83927476
POS_10445	Triacetylresveratrol	Stilbenes	3.560091769
POS_8204	Coumaric acid O-glucoside	Organooxygen compounds	0.382974237
POS_9307	beta-Gentiobiose	Organooxygen compounds	4.659777622
POS_392	Choline	Organonitrogen compounds	1.177553971
POS_19502	Chaetoglobosin C	Cytochalasans	0.573107415
POS_6314	Fluperlapine	Benzazepines	8.932526956
POS_20116	4-[4-Hydroxy-2,2,6-trimethyl-6-[[6-O-[(2S,3R,4R)-tetrahydro-3,4-dihydroxy-4-(hydroxymethyl)-2-furanyl]-beta-D-glucopyranosyl]oxy]cyclohexylidene]-3-buten-2-one	Prenol lipids	0.54999137
POS_7459	5-[(1S,4aS,8aS)-Decahydro-5,5,8a-trimethyl-2-methylene-1-naphthalenyl]-3-methyl-, (2E)-2-penten-1-ol	Prenol lipids	0.882350909
POS_13871	Vitamin E	Prenol lipids	0.377348737
